# Supplementary material for: Impact of Sample Preparation Methods on Single-Cell X-ray Microscopy and Light Elemental Analysis Evaluated by Combined Low Energy X-ray Fluorescence, STXM and AFM
Source: Molecules. 2023 Feb 20;28(4):1992. doi: 10.3390/molecules28041992 (PMC9962160; doi:10.3390/molecules28041992)
Supplement: Supplementary file 1 [file molecules-28-01992-s001.zip › molecules-2095087-supplementary.pdf]

molecules

MDPI

Supporting Information

Impact of Sample Preparation Methods on Single-Cell X-ray Microscopy and Light Elemental Analysis Evaluated by Combined Low Energy X-ray Fluorescence, STXM and AFM

Lucia Merolle [1], Lorella Pascolo [2], Luisa Zupin [2], Pietro Parisse [3,4], Valentina Bonanni [4], Gianluca Gariani [4], Sasa Kenig [5], Diana E. Bedolla [4,6], Sergio Crovella [7], Giuseppe Ricci [2,8], Stefano Iotti [9,10], Emil Malucelli [9], George Kourousias [4] and Alessandra Gianoncelli [4,*]

1 AUSL-IRCCS di Reggio Emilia, Transfusion Medicine Unit, Reggio Emilia, 42123, Italy
2 IRCCS Burlo Garofolo, Institute for Maternal and Child Health, Trieste, 34137, Italy
3 Consiglio Nazionale delle Ricerche, Istituto Officina dei Materiali, Trieste, 34149, Italy
4 Elettra—Sincrotrone Trieste S.C.p.A., 34149 Basovizza, 34149 Trieste, Italy
5 Faculty of Health Sciences, University of Primorska, Polje 42, 6310 Izola, Slovenia
6 Area Science Park, Padriciano 99, 34149 Trieste, Italy
7 Biological Science Program, Department of Biological and Environmental Science, College of Arts and Sciences, Qatar University, Doha 2713, Qatar
8 Department of Medical, Surgical, and Health Sciences, University of Trieste, 34149 Trieste, Italy
9 Department of Pharmacy and Biotechnology, University of Bologna, 40126 Bologna, Italy
10 National Institute of Biostructures and Biosystems, 00136 Rome, Italy
* Correspondence: alessandra.gianoncelli@elettra.eu

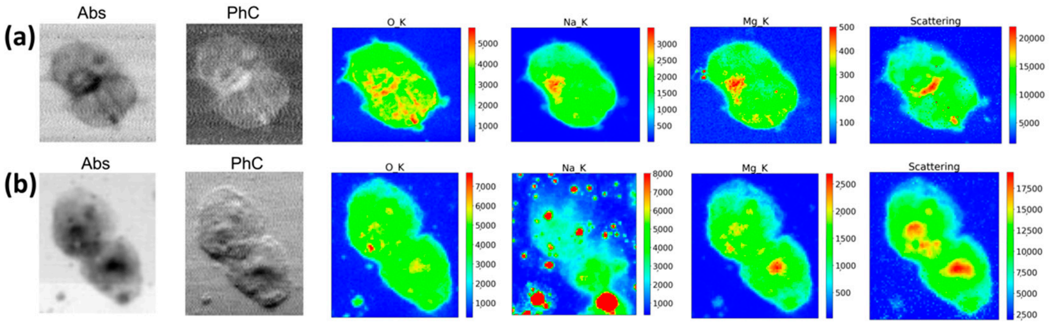

Figure S1. Absorption (Abs) and differential phase contrast (PhC) images, together with O, Na, Mg and scattering maps of HEK-293 cells fixed with different methods, a) 1:1 MeOH/C_3H_6O washed; b) and 70% Ethanol washed. Cells were fixed with the organic solvents for 3 min at -20°C, excess fixative was removed and samples were left to dry. In the washed conditions, excess fixative was removed, sample were quickly rinsed in DI water and then left to dry.

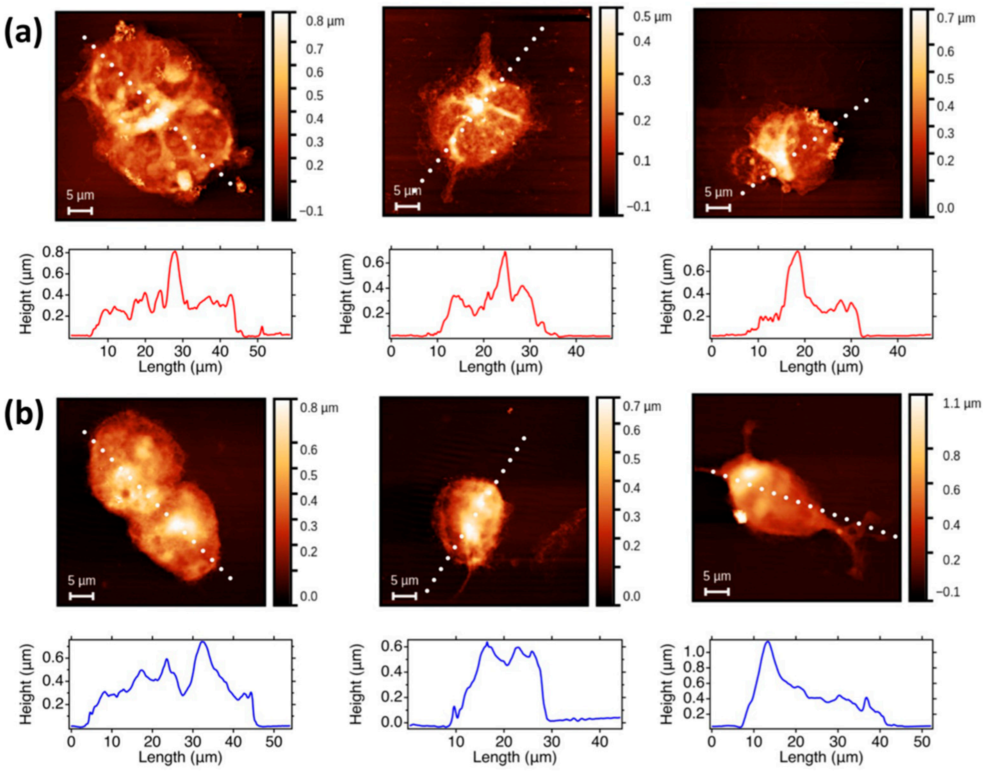

Figure S2. AFM images with corresponding surface profiles collected on a selection of a) 1:1 MeOH/C_3H_6O washed cells and of b) 70% Ethanol washed cells. The first column refers to the same cells shown in Figure S1.

Descriptive statistics

Oxygen fluorescence counts were used to asses precision of X-ray measurements related to different fixation methods. One-way ANOVA with Tukey's multiple comparisons test was performed to determine statistical significance statistical analysis was performed using GraphPad Prism 8.4.2 (GraphPad Software Inc., CA, USA).

Table S1. HEC-1-A descriptive analysis.

	**EtOH**	**PFA 2%**	**PFA 3.7%**
Number of values	6	6	6
Minimum	3670	2840	3550
25% Percentile	3670	2840	3550
Median	**3860**	**2900**	**3840**
75% Percentile	4130	4020	4260
Maximum	4130	4020	4260
Range	**460**	**1180**	**710**
Mean	**3887**	**3253**	**3883**
Std. Deviation	206	594	319
Std. Error of Mean	84	242	10
Coefficient of variation	5.95%	18.37%	9.20%

Table S2. Spermatozoa descriptive analysis.

	EtOH	PFA 2%	PFA 3.7%
**Number of values**	8	6	7
**Number of cells**	21	28	30
**Minimum**	3500	3880	3800
**25% Percentile**	3595	4285	3890
**Median**	**3985**	**4540**	**4350**
**75% Percentile**	4233	4685	5350
**Maximum**	4740	4790	5890
**Range**	**1240**	**910,0**	**2090**
**Mean**	**3975**	**4470**	**4583**
**Std. Deviation**	417	317	785
**Std. Error of Mean**	147	129	296
**Coefficient of variation**	10.51%	7.11%	17.14%
**Sum**	31800	26820	32080

Table S3. HEK-293 descriptive analysis.

	EtOH	PFA 2%	PFA 3.7%	MeOH/Ac.	Cryo-fixed
**Number of values**	5	5	5	4	6
**Minimum**	8152	8765	8981	17118	18582
**25% Percentile**	8152	8765	8981	17118	18582
**Median**	**9000**	**9231**	**10220**	**18341**	**19909**
**75% Percentile**	10369	9697	10685	19564	33182
**Maximum**	11738	9697	11149	19564	33182
**Range**	**3586**	**932**	**2168**	**2446**	**14600**
**Mean**	**9208**	**9138**	**10117**	**18341**	**23891**
**Std. Deviation**	1476	538	1088	1412	7221
**Std. Error of Mean**	660	269	628	706	2948
**Coefficient of variation**	16.03%	5.58%	9.48%	7.70%	30.23%

Table S4. HEK-293 Student's t-test analysis.

Tukey's Comparisons	Mean Diff.	Significant?	Summary	Adjusted p-value
EtOH vs. Cryo-fixed	-14683	Yes	*	0,0202
PFA 2% vs. Cryo-fixed	-14753	Yes	*	0,0368
PFA 3.7% vs. Cryo-fixed	-13981	Yes	*	0,0243

*P < 0.05.

Table S5. Comparison between AFM, STXM and LEXRF.

Technique	Attenuation length @ 1500eV	Chemical Sensitivity	Lateral Resolution	Vertical Resolution	Measurement time per pixel	Sample environment
**AFM**	NA	NA	80-100nm	0.5 nm	20-40 ms	Air
**STXM**	5um	NA	200-1000nm	NA	10-20ms	Vacuum
**LEXRF**	0.2um for O, 1um for Na, 3um for Mg emissions	0.2um for O, 1um for Na, 3um for Mg emissions	200-1000nm but for Signal/noise ratio reasons >400nm	NA	1-5 s	Vacuum
